# Supplementary material for: A set of multi-entry identification keys to African frugivorous flies (Diptera, Tephritidae)
Source: Zookeys. 2014 Jul 24;(428):97–108. doi: 10.3897/zookeys.428.7366 (PMC4143993; doi:10.3897/zookeys.428.7366)
Supplement: Supplementary material 6 — Key to Ceratitis [file zookeys-428-097-s006.zip › SF6_ZooKeys_key to Ceratitis/key/SF6_ZooKeys_key to Ceratitis/Media/Html/Ceratitis catoirii.htm]

Ceratitis (Ceratitis) catoirii Guérin-Méneville


***Ceratitis*** **(*Ceratitis*) *catoirii*
Guérin-Méneville**

*Ceratitis
catoirii*
Guérin-Méneville, 1843: 197.

Body
length: 5.25 (4.65-5.55) mm; wing length: 5.26 (4.70-5.55) mm.

Male

Head.
Antenna pale yellow. Third antennal segment twice as long as second segment. Arista
almost bare, at most with very short hairs on base. Frons pale yellow; with
short scattered hairs which are largely the same colour as frons. Frontal
bristles yellow, weakly developed; ocellar and orbital bristles pale or dark;
lower orbital modified, about as long as arista, apical end white and broadly
spatulate shaped; upper orbital weakly developed. Face yellow-white. Genal
bristle pale; genal setulae pale, weakly developed. Postocellar and outer
vertical pale.

Thorax.
Postpronotum yellow-white, with distinct black spot. Mesonotum: ground colour
black, microtrichiae pattern silvery with ashgrey shine, spots black except
sutural yellow-white spots, prescutellar yellow or yellow-white markings
separate or merged. Scapular setae pale. Scutellum yellow-white, basally with
two dark merged spots, apically with three merged spots, only slightly incised.
Anepisternum pale yellow, lower half darker yellow but indistinct, hairs in
lower half also slightly darker yellow. 

Legs
yellow; setation typical for subgenus, mainly pale especially on femora. Fore
femur posteriorly with bush of longer orange-coloured hairs along entire
length, posterodorsal hairs longer. Anteroventrally row of longer hairs, as
long as posterodorsal hairs; ventral spines yellow-orange. Mid femur with row
of long hairs anteroventrally. Hind femur with longer hairs dorsally and
ventrally on apical fourth. 

Wing.
Marginal band with clear and complete interruption; cubital band joined with
discal band; medial band free; crossvein r-m at middle of discal cell. Vein R1
beyond or equal with crossvein r-m. Crossvein dm-cu straight.

Abdomen.
Yellow. Setation and banding typical for subgenus.

Female

As
in male except for the following characters:  Third antennal segment darker,
yellow to yellow-orange. Hairs on arista slightly more developed. Frons yellow,
occasionally more palish; with short hairs which are distinctly darker than
frons. All cephalic bristles dark, except postocellar and outer vertical.
Orbitals not modified, frontals well developed. Genal bristle pale or dark;
genal setulae dark, strongly developed. Sometimes few dark hairs in lower half
of anepisternum. Scapular setae rarely black. Legs in general with darker
pilosity; no feathering; ventral spines on fore femur black. Wings, medial band
occasionally touching marginal. Crossvein dm-cu posteriorly more inwards than
anteriorly. Oviscape shorter than abdominal terga.

(Description
after De Meyer, 2000)
